# Genomic insights into antibiotic resistance, virulence traits and phylogenetic lineages of 141 clinical *Helicobacter pylori* isolates from Eastern China

**DOI:** 10.3389/fcimb.2025.1726881

**Published:** 2026-01-05

**Authors:** Pingping Wang, Jing Zhao, Bin Lv, Xiaohan Zhang, Ming Li, Shenglan Chen, Bingjie Fan, Wenhong Wang

**Affiliations:** 1 Department of Clinical Laboratory, Affiliated Taizhou Second People’s Hospital of Yangzhou University, Taizhou, Jiangsu, China; 2 Department of Gastroenterology, The First Affiliated Hospital of Zhejiang Chinese Medical University, Hangzhou, Zhejiang, China; 3 Cowin Medical Laboratory (Taizhou), Taizhou, Jiangsu, China; 4 Department of Laboratory Medicine, School of Medicine, Jiangsu University, Zhenjiang, Jiangsu, China

**Keywords:** antibiotic resistance, *Helicobacter pylori*, phylogenetic lineages, virulence genotypes, whole-genome sequencing

## Abstract

**Objective:**

To characterize antibiotic resistance, virulence genotypes and phylogenetic lineages of *Helicobacter pylori* (*H. pylori*) isolates from Eastern China and identify resistance/virulence-associated genetic variants.

**Methods:**

Whole-genome sequencing (WGS) and phenotypic antimicrobial susceptibility testing (AST) for 6 antibiotics were performed on 141 *H. pylori* isolates from Hangzhou, China. Genetic analysis (resistance mutations, virulence genotyping, phylogenetic tree) and the assessment of antibiotic resistance related phenotype-genotype concordance were conducted.

**Results:**

Metronidazole resistance was highest (85.1%), followed by levofloxacin (57.4%), clarithromycin (53.9%) and amoxicillin (21.3%); tetracycline and furazolidone resistance was low at 2.8% and 0.7%, respectively. Key resistance mutations included *23S rRNA* A2143G (clarithromycin, 92.91% phenotype-genotype concordance), *gyrA* N87K (levofloxacin, 89.13% concordance) and *pbp1A* 1785_1786insAGC (amoxicillin, 83.69% concordance). Dominant virulence genotypes: *cagA* ABD (86.26%), *vacA* s1-type (100%, 58.02% s1m2, 41.22% s1m1), *htrA* 171S (61.07%). 95.4% of strains clustered in hpEastAsia lineage; 3.8% in hpEurope, 0.76% in hpAsia2.

**Conclusion:**

*H. pylori* isolate in Eastern China show high resistance to common antibiotics and dominant high-virulence genotypes. WGS identifies key resistance markers, aiding targeted *H. pylori* treatment.

## Introduction

*Helicobacter pylori* (*H. pylori*) is a spiral-shaped, microaerophilic bacterium that colonizes the human stomach. Its discovery in the 1980s revolutionized our understanding of gastrointestinal diseases, as it has been firmly established as a major etiological agent for various gastric disorders, including chronic gastritis, peptic ulcers, and even gastric cancer (Jyh-Ming et al., 2025). The World Health Organization has classified *H. pylori* as a group I carcinogen, highlighting its significant role in the development of gastric malignancies (IARC, 1994).

The global prevalence of *H. pylori* infection remains high, with more than half of the world’s population estimated to be colonized (Chen et al., 2024). In China, the average individual-based infection rate was 40.66%, with 43.45% for adults and 20.55% for children and adolescents. Family-based infection rates ranged from 50.27% to 85.06% with an average rate of 71.21% (Zhou et al., 2023). However, the successful eradication of *H. pylori* has become increasingly challenging due to the rising prevalence of antibiotic resistance (Jearth et al., 2023). The emergence and spread of antibiotic-resistant *H. pylori* strains have led to treatment failures, longer treatment courses, and increased healthcare costs (Christian et al., 2025).

Understanding the genetic basis of *H. pylori* antibiotic resistance and virulence is crucial for the development of more effective treatment strategies (Retnakumar et al., 2025; Zhou et al., 2025). Antibiotic resistance in *H. pylori* is often associated with specific genetic mutations in genes related to drug-binding sites or metabolic pathways. For example, mutations in the *23S rRNA* gene are well-known to confer clarithromycin (CLA) resistance, while alterations in *gyrA* and *gyrB* genes are linked to levofloxacin (LEV) resistance (Jearth et al., 2023; Ng et al., 2023). In recent years, whole-genome sequencing (WGS) has been increasingly applied to dissect the genetic basis of antibiotic resistance and virulence in *H. pylori* by enabling high-resolution, multi-dimensional strain characterization. Unlike targeted sequencing or PCR-based methods, WGS captures the entire genomic landscape of *H. pylori*, allowing for: (1) comprehensive identification of resistance-associated mutations, including rare/novel variants that traditional approaches may miss (Wang et al., 2025b); (2) systematic profiling of virulence genotypes (e.g., cytotoxin-associated gene A (*cagA*), vacuolating cytotoxin A (*vacA*), and high-temperature requirement A (*htrA*)) to link genetic variation with pathogenic potential (Schubert et al., 2024; Zhu et al., 2025); (3) core genome-based phylogenetic analysis to clarify strain lineage origins, regional transmission, and evolutionary relationships with global populations (Kaisa et al., 2023; Retnakumar et al., 2025).

Despite advances in this field, critical knowledge gaps persist, particularly regarding the genetic characteristics of *H. pylori* in region-specific populations. Eastern China, with its high *H. pylori* infection burden and distinct clinical profiles, has seen relatively limited WGS-based investigations of local strains. To address this gap, the present study aimed to comprehensively characterize antibiotic resistance-associated genes and virulence factor genes in 141 clinical *H. pylori* isolates from this region. Through WGS and in-depth genetic analysis, we sought to identify potential novel genetic variants linked to antibiotic resistance and virulence, with the ultimate goal of providing insights to inform the development of more targeted, effective strategies for the treatment and prevention of *H. pylori*-related diseases.

## Materials and methods

### Clinical *H. pylori* isolates

The clinical *H. pylori* strains evaluated in this study were derived from a previous clinical trial performed in Hangzhou, an Eastern city of China, which was approved by the Ethics Committee of the first affiliated hospital of Zhejiang Chinese Medical University (2022-Q-005-01). In such clinical study, patients with dyspeptic symptoms who visited the first affiliated hospital of Zhejiang Chinese Medical University were recruited for endoscopy consecutively from January to September 2023 and gastric biopsy specimens were sampled from the antrum of each participant for the culturing and isolating of *H. pylori.* By using a standard culturing protocol, 171 *H. pylori* isolates were obtained and stored at -80°C (Hu, 2008). 141 isolates were recovered and tested of 6 antibiotics susceptibility in this study, which was approved by the Ethics Committee of the Taizhou Second People’s Hospital (KY2024-008-001). Since only gender and age information were retrieved and no personal identification information was involved, the informed consent form was waived.

### Phenotypic antibiotic susceptibility testing

Frozen *H. pylori* isolates were rapidly thawed in a 37 °C water bath for 1 min. A 300 μL aliquot of the thawed suspension was spread uniformly onto *H. pylori* culture plates (supplemented with 5% defibrinated sheep blood; Shanghai Kemajia Microbe Technology Co., Ltd., Shanghai, China), which were then incubated at 37 °C under microaerophilic conditions (5% O_2_, 10% CO_2_, 85% N_2_) for 2–3 days for optimal growth. *H. pylori* colonies were harvested with sterile saline-moistened swabs, suspended in physiological saline, and standardized to a McFarland turbidity of 2.0 ± 0.5 via nephelometer (Orient Chemical & Glass (Beijing) Technology Co., Ltd., Beijing, China) before uniform inoculation onto agar plates using a disposable sterile loop.

Antimicrobial susceptibility testing (AST) for CLA, LEV, amoxicillin (AMX), metronidazole (MTZ), and tetracycline (TET) was performed via E-test. Briefly, after bacterial lawn preparation, E-test strips (Wenzhou Kangtai Biotechnology Co., Ltd., Wenzhou, China) were aseptically applied to agar surfaces. Plates were incubated at 37 °C under the above microaerophilic conditions for 48 h; minimum inhibitory concentrations (MICs) were determined by reading the intersection of the inhibition ellipse with the strip scale. Resistance breakpoints followed EUCAST guidelines (Version 13.1, effective June 29, 2023) (Shang et al., 2015).

As no commercial E-test kits were available for furazolidone (FR), the Kirby-Bauer disk diffusion method was used for its AST. FR-impregnated disks (Wenzhou Kangtai Biotechnology Co., Ltd., Wenzhou, China) were placed on inoculated plates per standard procedures. After 48 h of microaerophilic incubation, inhibition zone diameters were measured with a vernier caliper: resistant (≤14 mm), intermediately susceptible (15–16 mm), susceptible (≥17 mm) (Zhong et al., 2021). *H. pylori* reference strain BNCC359033 (susceptible to above six antibiotics) was included as a quality control strain in each AST run.

### DNA extraction and WGS

Genomic DNA was extracted from *H. pylori* isolates using the genomic DNA extraction kit (Cat. No. CWY004M, Jiangsu Cowin Biotech Co., Ltd, Jiangsu, China) following the manufacturer’s standard protocols for Gram­negative bacteria. Briefly, bacterial cells were harvested from fresh cultures by centrifugation, resuspended in the provided lysis buffer, and subjected to proteinase K digestion at 56 °C until complete lysis. Genomic DNA was then purified through column-based adsorption and washing steps as specified in the kit instructions, and finally eluted in elution buffer. The concentration and purity of the extracted DNA were determined using a NanoDrop spectrophotometer (Thermo Fisher Scientific, USA) by measuring the absorbance ratios at 260/280 nm and 260/230 nm, with DNA integrity verified by 1% agarose gel electrophoresis.

For WGS of *H. pylori*, genomic DNA libraries were prepared using the Fast DNA Library Prep Set for Illumina & MGI (Cat. No. CW3045M, Jiangsu Cowin Biotech Co., Ltd, Jiangsu, China) following the manufacturer’s instructions. Briefly, genomic DNA was fragmented, end-repaired, and ligated with indexed adapters. Library quality and concentration were evaluated using a Qubit fluorometer (Thermo Fisher Scientific, USA), with the final library concentration adjusted to ≥10 nM to meet sequencing requirements. Qualified libraries were then loaded onto the MGISEQ-2000 platform (MGI Tech Co., Ltd, China) for paired-end sequencing (2×150 bp read length) following the standard operating procedures of the platform. Sequencing Quality Control data for all sequenced genomes including coverage, genome length, and N50 and L50 values, which are detailed in **Supplementary Table S1** in the **Supplementary Material**.

### Analysis of WGS data

The antibiotic resistance phenotypes of the 141 *H. pylori* strains were confirmed by culture-based susceptibility testing (gold standard). At the genomic level, we focused on 13 well-documented core resistance-associated genes of *H. pylori* (covering 6 classes of target antibiotics) and screened for resistance-related mutations through the following steps: ① Quality control and trimming of sequencing data were performed using FastQC v0.12.1 and Trim Galore v0.6.10; ② High-quality reads were aligned to the *H. pylori* 26695 reference genome (NC_000915.1) using the BWA-MEM v0.7.19 algorithm, and alignment results were standardized with the Picard 3.4.0 tool suite; ③ Variant calling was conducted with bcftools v1.22 to generate VCF files, and functional annotation of variants was performed using SnpEff v5.2f; ④ Using the results of susceptibility testing as the gold standard, strains were divided into resistant and susceptible groups. Chi-square test was applied to compare the mutation frequency differences at key loci of the 13 resistance-associated genes between the two groups, to screen for resistance-correlated mutations with statistical significance (*P* < 0.05).

In order to analyze the virulence genes, the assembled genomes were annotated using Prokka v1.14.6 to predict coding sequences (CDSs). To identify virulence genes such as *vacA* and *cagA*, the predicted CDSs were aligned against the Virulence Factors Database (VFDB) using BLASTp, with a set E-value cutoff of 1e-10 and sequence identity ≥ 95%. The EPIYA-A, EPIYA-B, EPIYA-C, and EPIYA-D motifs were defined based on the amino acid sequence surrounding the C-terminus of CagA. For the *vacA* gene, sequences were extracted using *vacA*-specific primers to determine its genotypes (s1, s2, m1, and m2). Additionally, high-quality sequencing reads were aligned to the *H. pylori* reference genome (NC_000915.1), and bcftools was employed to detect single nucleotide polymorphisms (SNPs) and small insertions/deletions (indels) in known virulence genes.

### Phylogenetic data analysis

After quality control and trimming of raw sequencing data using fastp v0.20.1, *de novo* assembly was performed with SPAdes 4.2.0. The assembly results (contigs) for each strain were saved in FASTA format, and assembly quality was evaluated using QUAST 5.3.0, including metrics such as N50, number of contigs, and genome coverage.

The assembled genomes, together with reference strain sequences downloaded from NCBI (see **Supplementary Table S2** in the **Supplementary Material**), were subjected to standardized annotation using Prokka 1.14.5 to predict functional elements including coding genes, rRNA, and tRNA. Based on the annotation results, pan-genome analysis was conducted using Roary v3.13.0 to determine the distribution characteristics of the core genome (genes shared by all strains) and accessory genome.

The core genome alignment results were extracted, and a phylogenetic tree was constructed using MEGA11 software with the Neighbor-Joining algorithm (parameters: 1000 bootstrap replicates, Tamura-Nei model). To enhance visual interpretation of the results, the generated Newick format tree file was uploaded to the iTOL online platform (https://itol.embl.de/) for customized visualization and labeling.

### Statistical analysis

Rates of the phenotypic resistance of *H. pylori* to 6 antibiotics were calculated. The mutation frequencies in phenotypically resistant strains to those in susceptible strains were compared to determine statistically significant mutations using the Chi-squared test or Fisher exact probability method, followed by the Bonferroni test to correct for multiple comparisons. The accordance rates and Cohen’s Kappa index were used to assess the agreement between phenotypic and genotypic antibiotic resistance. A kappa value of ≥0.75 indicates good consistency between the two methods. *P* < 0.05 indicated that the difference was statistically significant.

## Results

### Phenotypic antibiotic susceptibility testing

In this study, 141 isolates were successfully recovered and tested for susceptibility to 6 antibiotics. Antibiotic resistance of these isolates was determined by conventional culture and AST, as shown in **Table 1**. MTZ exhibited the highest resistance rate at 85.1%, followed by CLA and LEV, both with resistance rates exceeding 50%. The resistance rate to AMX was 21.3%. TET and FR showed relatively low resistance rates, with only 4 (2.8%) and 1 (0.7%) resistant isolate, respectively. No significant difference in antibiotic resistance was observed between genders. When age was stratified into three groups for comparison of resistance rates, only LEV resistance showed a significant difference among age groups, with the resistance rate in individuals over 30 years old being significantly higher than that in those under 30 years old.

**Table 1:** Demographic characteristics of 141 patients and phenotypic resistance of corresponding *H. pylori* isolates to 6 antibiotics.

Patient characteristic	No. of patients, n (%)	Antibiotic-resistant strain, *n* (%)					
		CLA	LEV	AMX	MTZ	TET	FR
Total	141(100)	76(53.9)	81(57.4)	30(21.3)	120(85.1)	4(2.8)	1(0.7)
Sex							
Male	68(48.2)	35(51.4)	38(55.9)	15(22.1)	59(86.8)	1(1.47)	1(1.47)
Female	73(51.8)	41(56.2)	43(58.9)	15(20.5)	61(83.6)	3(4.1)	0(0.0)
*P* Value		0.582	0.715	0.836	0.621	0.614	0.317
Age (yrs)							
≤30	16(11.3)	8(50.0)	4(25.0)	2(12.5)	12(75.0)	0(0.0)	0(0.0)
30-50	60(42.6)	28(46.7)	38(63.3)	9(15.0)	51(85.0)	1(1.7)	1(1.7)
≥51	65(46.1)	40(61.5)	39(60.0)	19(29.2)	57(87.7)	3(4.6)	0(0.0)
*P* Value		0.271	0.002*	0.254	0.463	0.331	0.562

CLR, clarithromycin; LEV, levofloxacin; AMX, amoxicillin; MTZ, metronidazole; TET tetracycline; FR, furazolidone. *, P < 0.05.

Analysis of single and multi-drug resistance patterns (**Table 2**) revealed that 11 isolates were susceptible to all 6 antibiotics, while no isolates were resistant to all 6 antibiotics. The most common single-drug resistance was to MTZ, found in 25 isolates. The predominant double-drug resistance patterns were LEV+MTZ, CLR+MTZ, and CLR+LEV. The most frequent triple-drug resistance pattern was CLR+LEV+MTZ, detected in 53 isolates, with all other triple-drug resistance patterns occurring in fewer than 20 isolates. For four-drug resistance, 15 isolates showed resistance to CLR+LEV+AMX+MTZ. TET and FR were excluded from the multi-drug resistance analysis due to their low resistance rates, with only 4 and 1 resistant isolates, respectively.

**Table 2:** Phenotypic resistance patterns of 141 *H. pylori* isolates.

Resistance pattern	Number of strains
Susceptible to all	11
Resistant to all	0
Mono resistant	
MTZ only	25
CLR only	2
LEV only	1
Double resistance	
LEV+MTZ	74
CLR+MTZ	68
CLR+LEV	58
AMX+MTZ	27
LEV+AMX	22
CLR+AMX	20
Multiple resistance	
CLR+LEV+MTZ	53
LEV+AMX+MTZ	19
CLR+LEV+AMX	18
CLR+AMX+MTZ	17
CLR+LEV+AMX+MTZ	15

Each resistance pattern is ordered from top to bottom by the proportion of strains in descending order. Only 4 strains were resistant to TET and 1 strain was resistant to FR; combinations involving TET or FR are not shown in this table.

CLR, clarithromycin; LEV, levofloxacin; AMX, amoxicillin; MTZ, metronidazole; TET tetracycline; FR, furazolidone.

**Figure 1:**
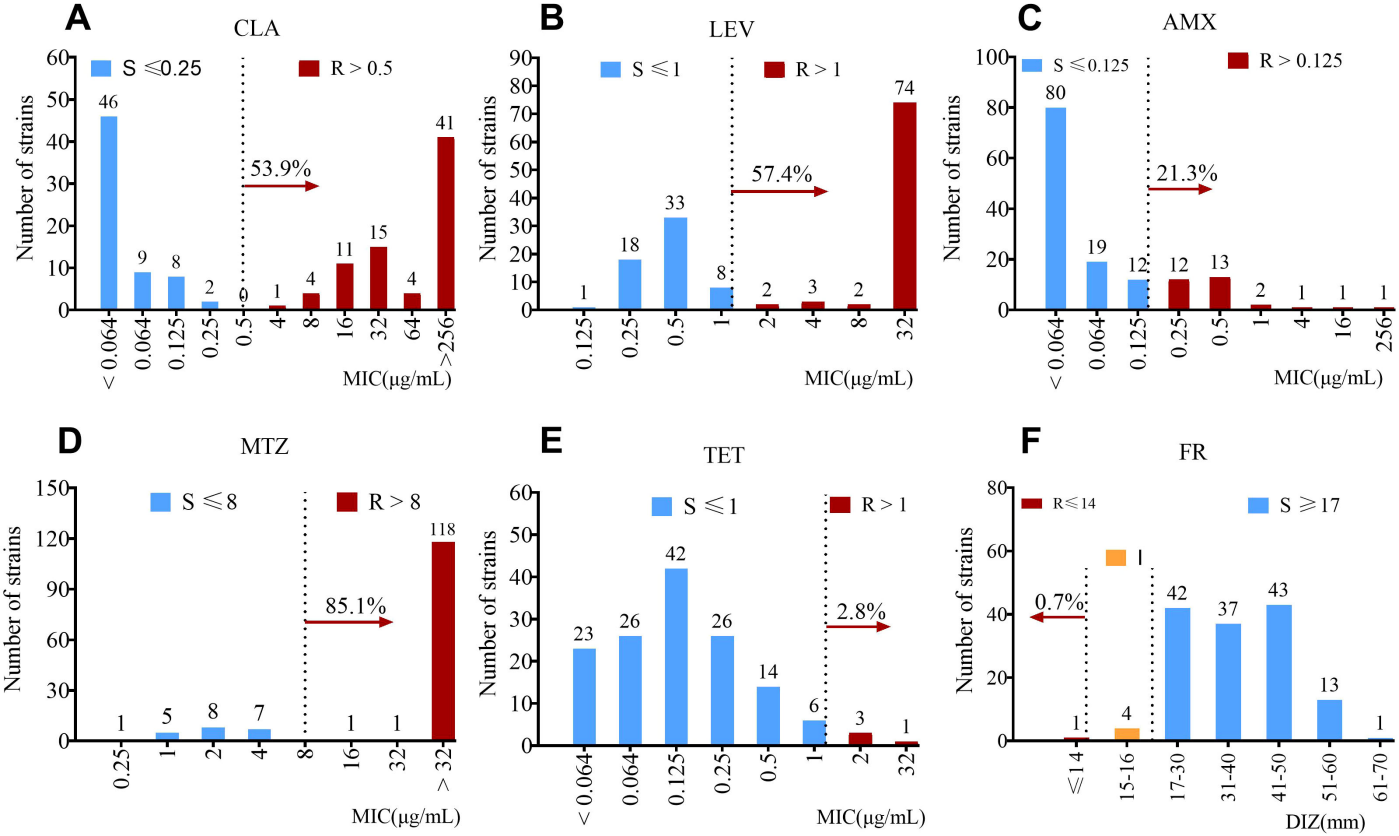
Distribution of minimum inhibitory concentrations (MICs) for strains exhibiting sensitivity or resistance to CLA, LEV, AMX, MTZ, TET and FR. Antibiotic resistance of 141 clinical isolates of **H. pylori** was determined. Resistance to CLA **(A)**, LEV **(B)**, AMX **(C)**, MTZ **(D)** and TET **(E)** was assessed using the E-test, following the breakpoints specified in the European Committee on Antimicrobial Susceptibility Testing (EUCAST) guidelines. For FR **(F)**, resistance was determined via the Kirby-Bauer (K-B) disk diffusion method, where a diameter of the inhibition zone (DIZ)≤14 mm was defined as resistance. The breakpoint for each antibiotic is indicated by a vertical dashed line in the figure. Abbreviations: “R” denotes resistant, “S” denotes susceptible, and “I” denotes intermediate susceptibility.

The distribution of MIC values for all 6 antibiotics across all isolates is shown in **Figure 1**. Only FR exhibited intermediate resistance in 4 isolates. Among resistant isolates, the majority displayed the highest MIC values for CLA, LEV, and MTZ. Notably, of the 120 MTZ -resistant isolates, 118 had MIC values >32 μg/mL. For susceptible isolates, most showed MIC values <0.064 μg/mL for CLA and AMX.

### Identification of genetic mutations associated with antibiotic resistance

A total of 141 clinical isolates of *H. pylori* were subjected to WGS in this study. Herein, 13 common resistance-associated genes, which have been reported in the literature to be associated with 6 types of antibiotics, were selected for analysis. Among these genes, 12 genes with statistically significant differences between resistant and susceptible strains before adjustment were presented in **Table 3**, while the remaining one (*porD*) was not included in the table as no statistically significant variations were observed even before adjustment. The analytical approach involved direct alignment of sequencing reads to the *H. pylori* standard strain 26695 reference genome. **Table 3** presents the mutation sites with statistically significant differences (un-adjusted) identified within these 12 genes between respective antibiotic-resistant and -susceptible strains. The Benjamini-Hochberg method was employed to correct for multiple testing errors, yielding adjusted *P*-values.

**Table 3:** Gene alteration differences between resistant and susceptible strains to 6 antibiotics in 141 whole-genome sequenced *H. pylori* isolates.

Antibiotics	Well established gene	Mutation type		Phenotypic resistance		*P* value	Adjusted *P* value
		CDS mutation	AA mutation	Resistant, n (%)	Sensitive, n (%)		
CLA	*23S rRNA*	T1644C		59 (78.67%)	59 (92.19%)	0.0326	1
		A2143G		67 (88.16%)	2 (3.08%)	0	0
		A2302G		0 (0.00%)	4 (6.15%)	0.0429	1
	*23S rRNA(A)*	1512_1513insC		9 (11.84%)	1 (1.54%)	0.0208	1
	*23S rRNA(B)*	T2317C		8 (10.53%)	1 (1.54%)	0.0381	1
		G1513C		7 (9.33%)	0 (0.00%)	0.0149	1
	*infB*	G1055A	R352H	2 (2.63%)	8 (12.31%)	0.0441	1
		T662C	V221A	61 (81.33%)	61 (96.83%)	0.0062	1
		554_555insTGTTAATAA	N185_A186insVNN	40 (53.33%)	23 (35.38%)	0.0413	1
		C110T	T37I	6 (8.00%)	0 (0.00%)	0.0303	1
LEV	*gyrA*	T261A	N87K	23 (29.49%)	0 (0.00%)	0	0
		T261G	N87K	10 (12.82%)	0 (0.00%)	0.0051	0.6885
		G271A	D91N	12 (15.38%)	1 (1.67%)	0.0074	0.7992
		G271T	D91Y	8 (10.26%)	0 (0.00%)	0.0132	1
		A272G	D91G	14 (17.95%)	1 (1.67%)	0.0021	0.378
		G1457A	R486H	3 (3.70%)	10 (16.67%)	0.0155	1
		2355_2356insTCC	E785_T786insS	0 (0.00%)	5 (8.33%)	0.0131	1
		2356_2358dup	T786dup	64 (82.05%)	32 (53.33%)	0.0018	0.486
	*gyrB*	G1033A	A345T	1 (1.23%)	6 (10.00%)	0.0419	1
AMX	*pbp-1A*	1785_1786insAGC	G595_V596insS	10 (33.33%)	3 (2.70%)	0	0
		1392_1393insAAA	K464dup	4 (13.33%)	1 (0.90%)	0.0074	1
		A1777G	T593A	9 (31.03%)	11 (10.00%)	0.0076	1
		1391_1392insAAA	K464dup	3 (10.00%)	0 (0.00%)	0.0089	1
		1390_1391insTAA	P463_K464insI	3 (10.00%)	0 (0.00%)	0.0089	1
		1786_1787insGCG	G595dup	8 (26.67%)	11 (9.91%)	0.0307	1
		1044del	I349fs	3 (10.00%)	32 (28.83%)	0.0345	1
		C1959A	S653R	2 (6.67%)	0 (0.00%)	0.0441	1
		A1553G	K518R	2 (6.67%)	0 (0.00%)	0.0441	1
		G1405A	V469M	2 (6.67%)	0 (0.00%)	0.0441	1
		1394_1395insAGA	K464_D465insE	2 (6.67%)	0 (0.00%)	0.0441	1
		1394_1395insAGT	D465delinsEV	2 (6.67%)	0 (0.00%)	0.0441	1
	*pbp2*	C1262T	T421I	3 (10.00%)	0 (0.00%)	0.0089	1
		69_70insGT	T24fs	7 (24.14%)	8 (7.21%)	0.0157	1
		A70G	T24A	17 (80.95%)	51 (53.68%)	0.0272	1
		G442A	A148T	6 (20.00%)	7 (6.31%)	0.0324	1
		A41G	Q14R	19 (67.86%)	50 (45.05%)	0.0358	1
		A70T	T24S	1 (3.70%)	21 (21.21%)	0.043	1
		1479_1480insC	S494fs	2 (6.67%)	0 (0.00%)	0.0441	1
	*Pbp3*	C337T	L113F	27 (96.43%)	85 (77.27%)	0.0273	1
		C1009T	P337S	8 (26.67%)	12 (10.91%)	0.0396	1
MTZ	*rdxA*	G278A	S93N	5 (4.17%)	4 (19.05%)	0.0283	1
		308_309insA	K104fs	0 (0.00%)	2 (9.52%)	0.0213	1
		316dup	R106fs	0 (0.00%)	2 (9.52%)	0.0213	1
		316_317insT	R106fs	0 (0.00%)	2 (9.52%)	0.0213	1
		C455T	A152V	8 (6.67%)	5 (23.81%)	0.0263	1
		652dup	*218fs	21 (17.50%)	0 (0.00%)	0.0431	1
	*recA*	G946A	A316T	0 (0.00%)	2 (9.52%)	0.0213	1
	*frxA*	T184A	L62I	3 (2.50%)	3 (14.29%)	0.0427	1
TET	*16S rRNA*	A928C		3 (75.00%)	19 (16.10%)	0.0121	1
FR	*oorD*	G22A	D8N	1 (25.00%)	0 (0.00%)	0.0355	1
		T149C	V50A	1 (25.00%)	0 (0.00%)	0.0355	1
		A261T	E87D	1 (25.00%)	0 (0.00%)	0.0355	1

CLR, clarithromycin; LEV, levofloxacin; AMX, amoxicillin; MTZ, metronidazole; TET tetracycline; FR, furazolidone; CDS, coding sequence; AA, amino acid. ‘*” indicates a frameshift mutation starting at amino acid position 218.

#### CLA

Two genes associated with CLA resistance were identified: the *23S rRNA* gene and the *infB* gene. A total of 1668 genetic variants (excluding synonymous mutations) were detected, among which 13 exhibited significant differences before adjustment. The A2143G mutation was consistently present across the two copies of the *23S rRNA* gene and remained statistically significant between CLA-resistant and -susceptible strains even after adjustment, indicating that it serves as an excellent marker for CLA resistance. Only two isolates carried the A2142G mutation, and no statistical difference was observed for this variant; notably, however, both isolates were phenotypically resistant. The 1512_1513insC and T2317C variants showed inconsistency between the two copies of the *23S rRNA* gene: the former was more common in copy A, while the latter was predominantly found in copy B. Nevertheless, no significant difference was detected between CLA-resistant and -susceptible strains after statistical adjustment. For the *infB* gene, four genetic variants showed *P* < 0.05 before adjustment; among these, the C110T variant was exclusively detected in CLA-resistant strains. However, all four variants lost statistical significance following adjustment.

#### LEV

For LEV resistance, the *gyrA* and *gyrB* genes were analyzed, and 540 genetic variants were identified. Among these, 9 variants showed significant differences before adjustment (all listed in **Table 3**). In the *gyrA* gene, mutations at position 261 resulted in an amino acid change of type N87K, while mutations at positions 271 and 272 led to amino acid changes of types D91N/Y/G. These mutation types exhibited highly significant statistical differences between LEV-resistant and -susceptible strains before adjustment. However, only the N87K variant remained statistically significant after adjustment. The A345T variant in the *gyrB* gene was primarily detected in LEV-susceptible strains.

#### AMX

Three genes associated with resistance *i.e.*, *pbp1A, pbp2*, and *pbp3* were analyzed in this study. A total of 739 genetic variants were detected, with 21 showing significant differences before adjustment (all listed in **Table 3**). After statistical adjustment, only the 1785_1786insAGC variant (corresponding to the amino acid change G595_V596insS) exhibited a statistically significant difference between AMX-resistant and -susceptible strains. Additionally, 9 genetic variants were exclusively present in AMX-resistant strains.

#### MTZ

The *rdxA*, *frxA*, and *recA* genes, which are associated with MTZ resistance, exhibited an extremely high number of genetic variants (up to 611). Despite this large number of variants, only 8 showed significant differences before adjustment (all listed in **Table 3**), and no variants remained statistically significant between resistant and susceptible strains after adjustment. Furthermore, the number of isolates carrying each of these mutations was extremely small, clearly indicating that these variants do not possess the characteristics of specific targets for MTZ resistance.

#### TET

The *16S rRNA* gene exhibited 607 genetic variants, among which 191 showed statistical differences between TET -resistant and -susceptible strains before adjustment. Given that resistance to TET is attributed to mutations at bases 926–928 in the *16S rRNA* gene, only mutations at these three bases are presented in **Table 3**. The results showed that only the A928C variant had a *P*-value < 0.05; specifically, 3 out of 4 phenotypically resistant isolates carried the A928C variant, though this difference lost statistical significance after adjustment. No isolates harbored mutations at base 926.

#### FR

Only one isolate exhibited FR resistance (inhibition zone diameter ≤ 14 mm). Regrettably, the sequencing depth (DP) of this isolate was < 100, leading to the classification of its sequencing as a failure. As an alternative approach, 4 isolates with intermediate susceptibility (inhibition zone diameter: 15–16 mm) were treated as resistant strains for comparative analysis with susceptible strains. We examined the *porD* and *oorD* genes to identify mutations reported to confer FR resistance. A total of 36 genetic variants were detected, among which 4 of *oorD* gene showed significant differences before adjustment (listed in **Table 3**). However, no variants remained statistically significant between resistant and susceptible strains after adjustment.

### Concordance between phenotypic and genotypic resistance profiles

Using the abovementioned genetic variants with established statistical differences between antibiotic-resistant and -susceptible strains as a basis, an additional analysis was conducted to quantify the concordance between phenotypic resistance profiles and genotypic variations. Owing to the limited number of meaningful characteristic genetic variants identified in this study, only three antibiotic-associated gene sets were included in the concordance analysis. Meanwhile, for this analysis, we also selected specific gene loci and mutation types that have been explicitly documented in previous literature, such as the *23S rRNA* A2142G variant (Nesrin and Bekir, 2022; Ho-Yu et al., 2023).

As shown in **Table 4**, the results demonstrated the following: The phenotypic resistance to CLA and LEV, corresponding to genotypic variations in the *23S rRNA* gene and *gyrA* gene respectively, both exhibited high concordance with their respective genotypic profiles. Genetic variations in the *pbp1A* gene were categorized into two types: one comprising well-documented common loci (e.g., amino acid substitutions at positions 562 and 593) reported in the literature (Zhu et al., 2025), and the other being the 1785_1786insAGC variant identified in the present study. Both types of *pbp1A* variations showed relatively high accordance rates with AMX phenotypic resistance; however, the Cohen’s Kappa coefficient for this correlation was low, indicating only weak to moderate agreement between *pbp1A* genotypic variations and AMX phenotypic resistance. Since our analysis identified no consistent resistance-linked genetic alterations for MTZ (and its point mutation patterns lacked regularity), MTZ was not included in the consistency analysis presented in **Table 4**.

**Table 4:** Agreement between phenotypic and genotypic resistance.

Antibiotics	Gene	Mutation type	Genotypic resistance	Phenotypic resistance		Accordance rate	Cohen’s Kappa	*P* value
				Resistant	Sensitive			
Clarithromycin	*23S rRNA*	A2142G; A2143G	Resistant	68	2	92.91%	0.858	<0.001
			Sensitive	8	63			
Levofloxacin	*gyrA*	N87A/K/I/Y/T; D91G/N/A/H/Y	Resistant	65	2	89.13%	0.783	<0.001
			Sensitive	13	58			
Amoxicillin	*pbp-1A*	N562D/H/Y; T593A/G/K/P/S	Resistant	10	14	76.26%	0.232	0.011
			Sensitive	19	96			
		1785_1786insAGC	Resistant	10	3	83.69%	0.386	<0.001
			Sensitive	20	108			

### Virulence genotyping

**Table 5** summarizes the genotypic profiles of three key virulence factors, *i.e*, *cagA, vacA* and *htrA*—among *H. pylori* isolates. Notably, 10 isolates were excluded from the virulence factor genotyping analysis due to invalid assembly results identified during quality control; consequently, all subsequent analyses were performed on 131 valid isolates. Virulence genotyping of each strain was detailed in **Supplementary Table S3** in the **Supplementary Material**.

**Table 5:** Virulence factors-based genotypes of 141 *H. pylori* isolates.

Virulence factors	Genotype	Isolates, n (%)
*cagA*	AB	2 (1.53)
	ABC	2 (1.53)
	ABD	113 (86.26)
	Other (AD,1; ADD,1; BD,1; ABCC,1)	4 (3.05)
	*cagA* negative	5 (3.82)
	Incomplete assembly	5 (3.82)
*vacA*	s1m1	54 (41.22)
	s1m2	76 (58.02)
	s2m1	0
	s2m2	0
	Incomplete assembly	1 (0.76)
*htrA* (S171L)	171S	80 (61.07)
	171L	51 (38.93)

Notes: In accordance with the quality control findings, 10 isolates were excluded due to their failure to produce valid assemblies. Consequently, the genotyping analysis of virulence factor genes presented herein was conducted on 131 isolates.

*cagA*, cytotoxin-associated gene A; *vacA*, vacuolating cytotoxin A; *htrA*, high-temperature requirement A; S, serine; L, leucine.

For the *cagA* gene, the ABD genotype was the most predominant, detected in 113 isolates (86.26%). The AB and ABC genotypes were less frequent; each identified in 2 isolates (1.53%). A small proportion of isolates carried rare *cagA* variants categorized as “Other” (4 isolates, 3.05%), including AD (1 isolate), ADD (1 isolate), BD (1 isolate), and ABCC (1 isolate). Additionally, 5 isolates (3.82%) were *cagA*-negative, and another 5 isolates (3.82%) had incomplete *cagA* assembly data, precluding definitive genotyping.

Regarding the *vacA* gene, all successfully genotyped isolates (130/131) belonged to two genotypes, with no s2m1 or s2m2 variants detected. The s1m2 genotype was the most common (76 isolates, 58.02%), followed by the s1m1 genotype (54 isolates, 41.22%). Only 1 isolate (0.76%) had incomplete *vacA* assembly, preventing genotype assignment.

For the *htrA* gene (focused on the S171L polymorphism), the wild-type 171S genotype was more prevalent than the variant 171L genotype. Specifically, 80 isolates (61.07%) carried the 171S allele, while 51 isolates (38.93%) harbored the 171L allele, with no missing or ambiguous genotypes observed for this polymorphism.

### Phylogenetic analysis

**Figure 2:**
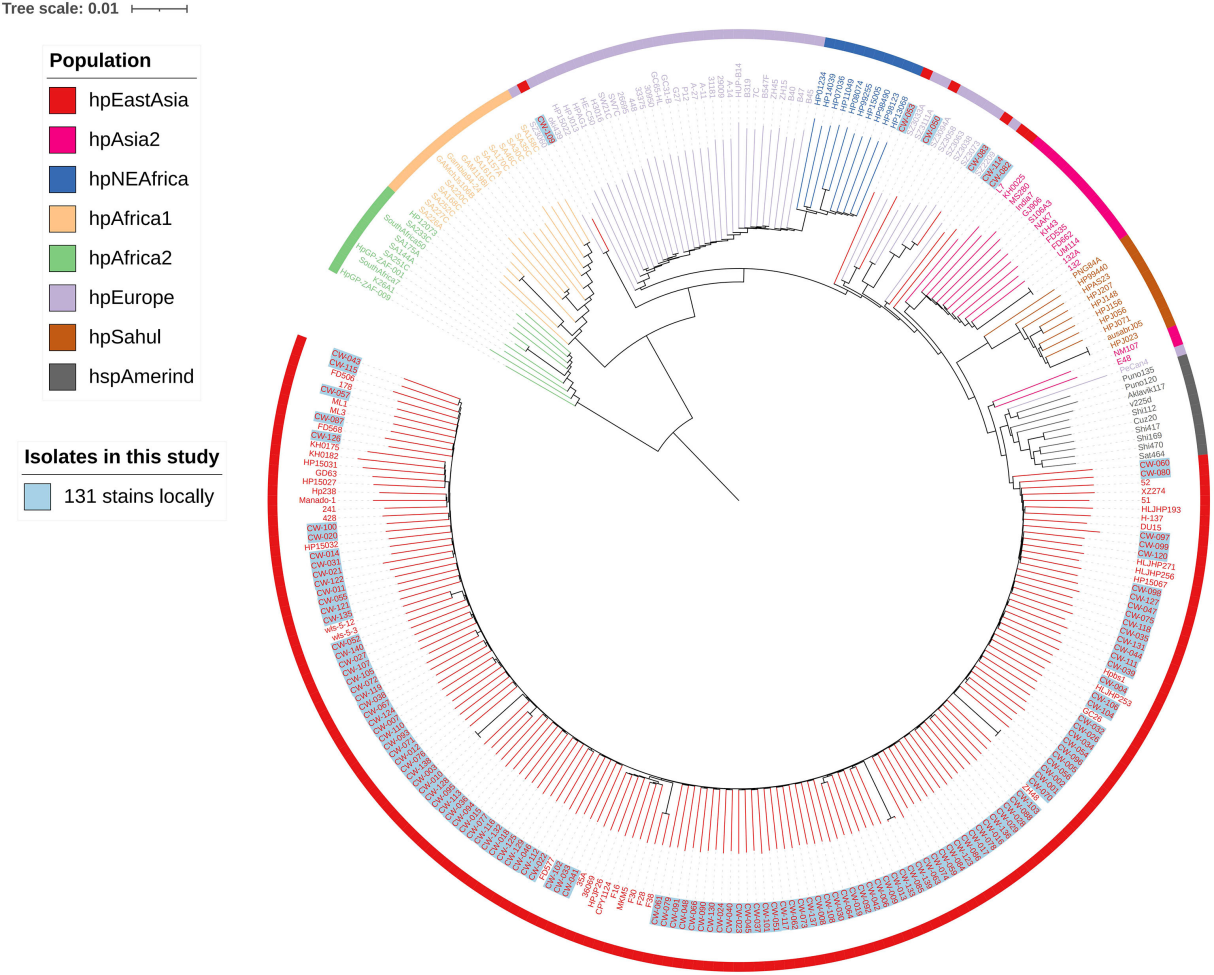
Phylogenetic tree of the isolated *H. pylori* strains. Based on the quality control results, 10 isolates could not be effectively assembled; thus, only 131 isolates were included in the phylogenetic tree analysis herein. The assembled genomes, together with reference strain sequences downloaded from NCBI, were annotated with Prokka 1.14.5 (to predict coding genes, rRNA, tRNA), followed by pan-genome analysis using Roary v3.13.0 to clarify core (shared by all strains) and accessory genome distribution. Core genome alignments were used to build a phylogenetic tree via MEGA11 (Neighbor-Joining, 1000 bootstraps, Tamura-Nei model); the Newick tree was visualized/labeled on ITOL (https://itol.embl.de/).

As depicted in the phylogenetic tree, *H. pylori* isolates were categorized into multiple well-defined populations, including hpEastAsia, hpAsia2, hpNEAfrica, hpAfrica1, hpAfrica2, hpEurope, hpSahul, and hspAmerind, with each clade distinctly differentiated by unique color coding for enhanced visual discrimination (**Figure 2**). Notably, the 131 locally sourced *H. pylori* strains in this study, which were marked with light blue labels, exhibited a striking phylogeographic clustering pattern: the vast majority (125/131, 95.4%) were tightly aggregated within the hpEastAsia population (highlighted in red), forming a monophyletic subclade with strong bootstrap support (≥85% at major nodes). This dominant clustering underscores a close genetic affinity between the local isolates and reference strains of East Asian origin, consistent with the well-documented geographic stratification of *H. pylori* populations. Of the remaining 6 isolates, 5 strains clustered within the hpEurope population. The remaining 1 isolate (CW-082) were phylogenetically affiliated with the hpAsia2 population, which is typically associated with South Asian and Central Asian *H. pylori* lineages.

## Discussion

Through WGS and phenotypic analysis of 141 clinical *H. pylori* isolates from Eastern China, this study obtained core findings in three aspects. For antibiotic resistance, MTZ (85.1%), CLA (53.9%), and LEV (57.4%) showed high resistance rates, with *23S rRNA* A2143G, *gyrA* N87K, and *pbp1A* 1785_1786insAGC identified as key resistance mutations. Phenotype-genotype concordance was high for CLA and LEV but low for AMX. In terms of virulence genotypes, the isolates were dominated by high-virulence profiles: *cagA* ABD (86.26%), *vacA* s1 subtype (100%, 58.02% s1m2, 41.22% s1m1), and the *htrA* S171L polymorphism displayed a slight 171S variant predominance: 61.07% (171S) versus 38.93% (171L). For phylogenetic lineages, 95.4% (125/131) of isolates clustered in the hpEastAsia population (bootstrap support ≥85%), while 3.1% (4 strains) belonged to hpEurope and 1.5% (2 strains) to hpAsia2.

A recent meta-analysis reported that the overall resistance rates of *H. pylori* in mainland China were 30.72% to CLA, 70.14% to MTZ and 32.98% to LEV; for AMX, TET, and FR, the rates were 2.41%, 2.53% and 1.54%, respectively (Zeng et al., 2024). A 2023 meta-analysis summarizing phenotypic resistance studies of *H. pylori* in Zhejiang province, all based on the disk diffusion method, reported that CLA resistance rates ranged from 12.56% to 33.86%, while LEV resistance rates varied between 28.69% and 44.88% (Chen et al., 2022). Additionally, Zhang et al. conducted a retrospective analysis of phenotypic antimicrobial susceptibility results from 9,430 cases in Taizhou, Zhejiang, and found the average CLA resistance rate was 23.99% and the average LEV resistance rate was 30.29% (Zhang et al., 2021). However, a 2024 study by Fang et al. reported significantly higher resistance rates in Hangzhou, Zhejiang: 79.17% for CLA and 64.58% for LEV, which were notably higher than those in our study (53.9% for CLA and 57.4% for LEV) and other studies conducted in this region (Fang et al., 2024). To account for the unexpectedly high resistance rates observed in their Hangzhou-based study, Fang et al. proposed that the widespread use of these antibiotics for non-*H. pylori* infections (such as respiratory and urinary tract infections) might be a key driver. Nevertheless, this discrepancy could not be fully explained by this factor alone; the selection of the study population and the relatively limited sample size are also plausible contributing variables.

In the same meta-analysis, the MTZ resistance rate in Zhejiang ranged from 80.15% to 96.79% (Chen et al., 2022), while Zhang et al. reported a rate of 93.72% in Taizhou, Zhejiang (Zhang et al., 2021). Within Hangzhou specifically, Fang et al. documented an MTZ resistance rate of 86.42%, which was comparable to our finding of 86.8%. Our study identified CLA+LEV+MTZ as the most prevalent triple-drug resistance pattern (53 isolates), accounting for over 60% of all multidrug-resistant strains. This multidrug resistance pattern is consistent with findings from studies in South Korea and Japan (Hong et al., 2024; Christian et al., 2025). Since 2020, CLR+LEV+MTZ resistance has become the dominant multidrug resistance type in these countries, underscoring the shared challenge faced by East Asian regions in addressing multidrug-resistant *H. pylori*.

No resistance to AMX, TET, or FR was reported in the 2023 meta-analysis (Chen et al., 2022). Zhang et al. documented an AMX resistance rate of merely 0.21% in Taizhou, Zhejiang (Zhang et al., 2021), whereas our study detected a significantly higher AMX resistance rate of 21.3% in Hangzhou, which was still lower in comparison to earlier reported in the same city *i.e.*, 37.50% (Fang et al., 2024). These findings indicate that AMX resistance is substantially higher in patients seeking care in Hangzhou than in non-Hangzhou populations, warranting caution regarding unsatisfactory eradication outcomes when selecting AMX-based dual therapy in clinical practice. Resistance to TET and FR remained extremely low overall, except for the 10.42% TET resistance rate in Hangzhou reported by Fang et al (Fang et al., 2024).

In summary, MTZ resistance rates are consistently extremely high across Zhejiang province, while TET and FR resistance rates are generally low, consistent with findings nationwide. Although the resistance rates of CLA, LEV, and AMX in our study were lower than those reported by Fang et al., they all exceeded the national average and the rates in other regions of Zhejiang, suggesting a marked increase in antibiotic resistance in Hangzhou that requires validation with expanded sample sizes. Additionally, LEV resistance is significantly elevated in patients over 30 years of age. Therefore, clinicians should inquire about patients’ age and medication history before treatment, perform AST when necessary, and avoid using antibiotics to which *H. pylori* is resistant to ensure eradication efficacy and mitigate the progression of antibiotic resistance.

Traditional techniques such as conventional PCR and Sanger sequencing are limited to known gene loci, making it difficult to comprehensively analyze the associations among drug resistance mutation profiles, virulence genotypes, and phylogenetic lineages, nor can they identify novel drug resistance markers or potential evolutionary patterns. WGS has overcome this limitation. In this study, we applied WGS to analyze 141 clinical *H. pylori* isolates from Hangzhou, yielding key findings in the field of resistance genetics.

Regarding CLA resistance, the *23S rRNA* gene A2143G mutation was identified as the absolute core driver: its detection rate reached 88.16% in resistant strains, compared with only 3.08% in susceptible strains, with an adjusted *P*-value approaching 0, indicating a strong association with CLA resistance. Further correlation between mutation information and MIC values showed that the majority of resistant strains carrying the A2143G mutation had MIC values >32 μg/mL, which was significantly higher than those of resistant strains without this mutation (MIC values mostly ranging from 4 to 16 μg/mL) and far exceeding the clinical resistance threshold for CLA (usually >0.5 μg/mL). These results confirm that the A2143G mutation is critical for high-level CLA resistance, consistent with conclusions from most global studies that this mutation impairs CLA binding by altering the spatial structure of the 50S ribosomal subunit, serving as the primary molecular marker for CLA resistance (Vincenzo et al., 2009; Tae Jun et al., 2010). The *23S rRNA* gene of *H. pylori* typically has two copies, a genetic redundancy feature that provides a unique molecular context for the accumulation and expression of resistance mutations. In this study, no difference in the A2143G mutation was observed between the two copies, suggesting that detecting either copy is sufficient for clinical testing. In contrast, mutations such as 1512_1513insC, T2317C, and G1513C showed discrepancies between the two copies; their impacts on resistance evolution and clinical efficacy require further verification.

The mechanism of AMX resistance primarily involves penicillin-binding proteins (PBPs), which mediate resistance by interfering with the transpeptidase activity required for cross-linking nascent peptidoglycan. After adjusted testing in this study, only the 1785_1786insAGC insertion variant in the *pbp1A* gene was found to be associated with AMX resistance: 10 out of 30 AMX-resistant strains (33.33%) carried this insertion variant. No other statistically significant alterations were detected in the *pbp1A*, *pbp2*, or *pbp3* genes, including the traditionally recognized variations at positions 562 and 593. Existing studies have confirmed that insertion variants in PBP1 can confer AMX resistance in bacteria such as *H. pylori*. Takeshi et al. analyzed clinically isolated AMX-resistant strains in South Korea and identified an amino acid insertion mutation in the *pbp1* gene, which was located in the C-terminal region containing the penicillin-binding module and further gene transformation experiments verified a causal relationship between this insertion variant and resistance (Takeshi et al., 2002). However, the direct association between the 1785_1786insAGC insertion variant identified in this study and AMX resistance still requires support from epidemiological data across more regions and functional validation studies.

MTZ resistance is thought to be highly associated with mutations in the *rdxA*, *recA*, and *frxA* genes. However, multiple recent WGS studies in China (covering regions such as Hainan, Tianjin, North China, and Zhejiang) have failed to identify characteristic genetic alterations that can be used to predict MTZ resistance (Fang et al., 2024; Lv et al., 2024; Tong et al., 2024; Wang et al., 2025a). Only Zhu et al. reported that the Y103H and S121D variants in *recA* differed between resistant and susceptible strains (Zhu et al., 2025). Consistent with these findings, our study also detected no meaningful MTZ resistance-associated genetic changes, and the point mutation patterns showed no consistent regularity. These results suggest that further exploration of molecular susceptibility markers for MTZ is needed, and constructing a multi-marker machine learning model may be an effective strategy superior to relying on single-base mutations for resistance prediction (Guo et al., 2022; Wang et al., 2025b). In addition, no resistance markers for TET or FR were identified in this study, which is presumably related to the small number of strains included for these two antibiotics and potential research bias.

Genotype-phenotype concordance analysis revealed that CLA resistance based on *23S rRNA* gene mutations and LEV resistance based on *gyrA* gene mutations could both distinguish phenotypic susceptibility from resistance well. This indicates that these mutations are excellent diagnostic markers, which can be used to develop corresponding diagnostic kits for clinical application. For AMX, although some *pbp1A* gene-related variants were identified, the overall concordance index was relatively low.

As the most critical virulence marker of *H. pylori*, CagA has EPIYA motif subtypes directly linked to pathogenic potential. The ABD subtype, containing multiple phosphorylation sites, can strongly activate host cell signaling pathways and significantly increase the risk of gastric cancer. Our results showed that the *cagA* ABD subtype was absolutely dominant in this study (86.26%), with AB and ABC subtypes accounting for only 1.53% each and *cagA*-negative strains merely 3.82%. This is consistent with the fact that the samples in this study are mainly of the hpEastAsia lineage, and also highly consistent with the characteristic of prevalent enrichment of high-virulence CagA subtypes in East Asian regions (Eastern China, Japan, South Korea) (Hayashi et al., 2020; Zhu et al., 2025).

The *vacA* genotype is determined by the combination of the signal region (s) and middle region (m). Among these, the s1 subtype is defined as a high-virulence phenotype due to its ability to efficiently secrete vacuolating toxin and induce cell apoptosis, whereas the s2 subtype exhibits significantly reduced pathogenicity as it lacks a signal peptide and thus cannot secrete the toxin. As shown in **Table 5**, *vacA* subtypes in the strains of this study were 100% s1-type (s1m1: 41.22%; s1m2: 58.02%), with no s2 subtype detected. This result is consistent with findings from studies in Tianjin, Shandong, Beijing, Shanghai and other regions of China, where the proportion of the s1 subtype exceeded 90% (Wang et al., 2023; Li et al., 2024; Xue et al., 2024; Men et al., 2025); notably, the proportion of the s1 subtype was also 100% in the Shandong study. All these proportions are significantly higher than those in European regions (s1 subtype: 60%–70%) (Zhu et al., 2025), reflecting that *H. pylori* strains in China generally exhibit strong vacuolating toxin activity.

The 171S/L mutation in *htrA* is a unique bacterial cancer-associated single nucleotide polymorphism (SNP) and a novel potential biomarker for predicting infection risk in *H. pylori* infections. As a virulence factor with both protease and adhesion functions, HtrA is closely associated with gastric cancer development (Irshad et al., 2023; Bodo et al., 2024). Since this SNP was newly identified in 2023, there are few reports on its distribution in natural *H. pylori* populations to date. This study represents the first report on the proportion of the 171S and 171L *htrA* variants in China, with the 171S variant accounting for 61.07% and the 171L variant for 38.93%. In Mongolia, another East Asian region, a previous study reported that the 171S variant accounted for 84.3% and the 171L variant for 15.7%, with the proportion of the more pathogenic 171S variant being higher than that observed in our study (Saruuljavkhlan et al., 2023). Further studies across more regions worldwide are needed to clarify the polymorphic characteristics of the *HtrA* 171 locus in different global populations and across various gastric disease states. Through evolutionary analysis, Linz et al. proposed that all *H. pylori* strains carried the 171L *HtrA* variant during the out-of-Africa migration of humans; the 171S mutation originated in Asia and subsequently spread globally (Bodo et al., 2024). This finding may partially explain why the proportion of the 171S variant was higher than that of the 171L variant in both our study and the Mongolian study.

Phylogenetic tree results showed that 95.4% (125/131) of the strains in this study were tightly clustered within the hpEastAsia lineage. This is highly consistent with the geographical stratification characteristics of *H. pylori* populations in East Asian regions (China, Japan, South Korea) (Waskito and Yamaoka, 2019; Kaisa et al., 2023; Lv et al., 2024). The absolute dominance of the hpEastAsia lineage in this study confirms the “locally originated” evolutionary pattern of *H. pylori* populations in Hangzhou, Zhejiang province, which echoes the findings from studies in East China regions such as Shanghai and Nanjing in China, where the proportion of the hpEastAsia lineage exceeded 90% (Zhou et al., 2022; Zhu et al., 2025). In the phylogenetic tree, 3.8% (5/131) of the strains were clustered within the hpEurope lineage and nested in the subclade composed of reference strains from Western Europe and the Mediterranean region. Additionally, 0.76% (1/131) of the strains were assigned to the hpAsia2 lineage, which is typically associated with strains from South Asia and Central Asia. The presence of the hpEurope and hpAsia2 lineages is generally closely linked to cross-regional human migration. As an economic hub in the Eastern coastal region of China, Hangzhou (Zhejiang province) has witnessed increasingly frequent commercial, tourism, and study-abroad exchanges with European countries, South Asia, and Central Asia in recent years. It is plausible that individuals involved in such exchanges may have introduced overseas *H. pylori* strains into the local area through “oral-oral transmission” or “fecal-oral transmission”.

This study provides data support for the analysis of genomic characteristics of *H. pylori* in a regional population, but has the following key limitations: First, the samples were only collected from patients attending the department of Gastroenterology at a single tertiary hospital in Hangzhou, without including asymptomatic individuals, cases from primary healthcare institutions, or specific age groups (e.g., children). This may lead to an overestimation of the regional drug resistance rate due to the relatively high history of antibiotic exposure among the enrolled patients. Second, genomic analysis was limited to descriptive statistics, and no “genotype-phenotype” prediction model was constructed. Additionally, the synergistic mechanism underlying the “high virulence + high drug resistance” phenotype of the hpEastAsia lineage was not explored.

In future studies, it will be necessary to expand the sample coverage, integrate longitudinal follow-up data and *in vitro* experiments, and deepen mechanistic research to improve the practical utility of the findings.

## Data Availability

The datasets and materials used and/or analyzed during the current study are available from the corresponding author upon reasonable request. Data are deposited in National Microbiology Data Center (NMDC) with accession numbers NMDC10020204 (https://nmdc.cn/resource/genomics/project/detail/NMDC10020204).

## References

[B1] International Agency for Research on Cancer (IARC) . (1994). Schistosomes, liver flukes and *Helicobacter pylori*. IARC. Monogr. Eval. Carcinog. Risks. Hum. 61, 177–240. 7715068 PMC7681621

[B2] Bodo L. Heinrich S. Nicole T. Steffen B. (2024). Cancer-associated SNPs in bacteria: lessons from *Helicobacter pylori*. Trends Microbiol. 32, 847–857. doi: 10.1016/j.tim.2024.02.001, PMID: 38485609

[B3] Chen J. Li P. Huang Y. Guo Y. Ding Z. Lu H. (2022). Primary antibiotic resistance of *helicobacter pylori* in different regions of China: A systematic review and meta-analysis. Pathogens 11, 786–800. doi: 10.3390/pathogens11070786, PMID: 35890031 PMC9316315

[B4] Chen Y.-C. Malfertheiner P. Yu H.-T. Kuo C.-L. Chang Y.-Y. Meng F.-T. . (2024). Global prevalence of *Helicobacter pylori* infection and incidence of gastric cancer between 1980 and 2022. Gastroenterology 166, 605–619. doi: 10.1053/j.gastro.2023.12.022, PMID: 38176660

[B5] Christian S. Jyh-Ming L. Mohamed A. Jan B. Christian C. N. Luiz Gonzaga C. . (2025). *Helicobacter pylori* antibiotic resistance: a global challenge in search of solutions. Gut 74, 1561–1570. doi: 10.1136/gutjnl-2025-335523, PMID: 40645767

[B6] Fang Y. Jiang S. Zhou X. Zhou W. Jiang X. Chen L. . (2024). Whole-genome sequencing analyses and antibiotic resistance situation of 48 *Helicobacter pylori* strains isolated in Zhejiang, China. Gut pathogens. 16, 62–74. doi: 10.1186/s13099-024-00656-2, PMID: 39444024 PMC11515586

[B7] Guo Z. Tian S. Wang W. Zhang Y. Li J. Lin R. (2022). Correlation analysis among genotype resistance, phenotype resistance, and eradication effect after resistance-guided quadruple therapies in refractory *Helicobacter pylori* infections. Front. Microbiol. 13. doi: 10.3389/fmicb.2022.861626, PMID: 35330762 PMC8940283

[B8] Hayashi H. Inoue J. Oyama K. Matsuoka K. Nishiumi S. Yoshida M. . (2020). Detection of novel amino acid polymorphisms in the east asian cagA of *Helicobacter pylori* with full sequencing data. Kobe. J. Med. Sci. 66, E22–E31., PMID: 32814754 PMC7447099

[B9] Hong T. C. El-Omar E. M. Kuo Y. T. Wu J. Y. Chen M. J. Chen C. C. . (2024). Primary antibiotic resistance of Helicobacter pylori in the Asia-Pacific region between 1990 and 2022: an updated systematic review and meta-analysis. Lancet Gastroenterol. Hepatol. 9, 56–67. doi: 10.1016/s2468-1253(23)00281-9, PMID: 37972625

[B10] Ho-Yu N. Wai K L. Ka-Shing C. (2023). Antibiotic resistance, susceptibility testing and stewardship in *helicobacter pylori* infection. Int. J. Mol. Sci. 24. doi: 10.3390/ijms241411708, PMID: 37511471 PMC10380565

[B11] Hu F. L. Z. (2008). Youmenluoganjun Ganran de Jichu yu Linchuang (Basic and Clinical Aspects of Helicobacter pylori Infection). 3rd edn (Beijing, China: China Science and Technology Press).

[B12] Irshad S. Nicole T. Bodo L. Manfred R. Michael V. Alfred Chin-Yen T. . (2023). A single-nucleotide polymorphism in *Helicobacter pylori* promotes gastric cancer development. Cell Host Microbe 31, 1345–1358. doi: 10.1016/j.chom.2023.06.016, PMID: 37490912

[B13] Jearth V. Rath M. Chatterjee A. Kale A. Panigrahi M. (2023). Drug-resistant *Helicobacter pylori*: diagnosis and evidence-based approach. Diagnostics (Basel Switzerland). 13, 2944–2958. doi: 10.3390/diagnostics13182944, PMID: 37761310 PMC10528400

[B14] Jyh-Ming L. Peter M. Tzu-Chan H. Hsiu-Chi C. Kentaro S. Shailja S. . (2025). Screening and eradication of *Helicobacter pylori* for gastric cancer prevention: Taipei Global Consensus II. Gut 74, 1767–1791. doi: 10.1136/gutjnl-2025-336027, PMID: 40912906

[B15] Kaisa T. Zilia Y M.-R. Difei W. Santiago S.-M. Rajiv B. A. Silvia G. . (2023). The *Helicobacter pylori* Genome Project: insights into *H. pylori* population structure from analysis of a worldwide collection of complete genomes. Nat. Commun. 14, 8184–8199. doi: 10.1038/s41467-023-43562-y, PMID: 38081806 PMC10713588

[B16] Li C. He L. Wang A. Chen S. Fu P. Wang C. (2024). Antibiotic resistance and virulence genes in *Helicobacter pylori* strains isolated from children in Shanghai, China, (2019-2022). Int. J. Med. Microbiol. 315, 151622–151628. doi: 10.1016/j.ijmm.2024.151622, PMID: 38776570

[B17] Lv Y. Li D. Zhang D. Chen S. Chen R. Wang Y. . (2024). *Helicobacter pylori* resistance in Hainan province, China: investigating phenotypes and genotypes through whole-genome sequencing. Front. Cell. Infect. Microbiol. 14. doi: 10.3389/fcimb.2024.1505166, PMID: 39742338 PMC11685075

[B18] Men C. J. Shan S. X. Ou Z. Y. Fu H. L. Wang B. Tong Y. . (2025). Detection of virulence genes in *Helicobacter pylori* and its correlation with drug resistance by polymerase chain reaction. Eur. J. Med. Res. 30, 523–533. doi: 10.1186/s40001-025-02792-0, PMID: 40555996 PMC12186420

[B19] Nesrin G. Bekir K. (2022). Detection of A2143G, A2142C, and A2142G point mutations with real-time PCR in stool specimens from children infected with *Helicobacter pylori*. Diagnostics (Basel). 12, 12. doi: 10.3390/diagnostics12092119, PMID: 36140521 PMC9497693

[B20] Ng H. Leung W. Cheung K. J. (2023). Antibiotic resistance, susceptibility testing and stewardship in infection. Int. J. Mol. Sci. 24, 11708–11740. doi: 10.3390/ijms241411708, PMID: 37511471 PMC10380565

[B21] Retnakumar R. J. Chettri P. Lamtha S. C. Sivakumar K. C. Dutta P. Sen P. . (2025). Genome-wide accumulations of non-random adaptive point mutations drive westward evolution of *Helicobacter pylori*. BMC Microbiol. 25, 229–248. doi: 10.1186/s12866-025-03944-2, PMID: 40263995 PMC12013172

[B22] Saruuljavkhlan B. Alfaray R. I. Oyuntsetseg K. Gantuya B. Khangai A. Renchinsengee N. . (2023). Study of *Helicobacter pylori* Isolated from a High-Gastric-Cancer-Risk Population: Unveiling the Comprehensive Analysis of Virulence-Associated Genes including Secretion Systems, and Genome-Wide Association Study. Cancers (Basel). 15, 4528–4551. doi: 10.3390/cancers15184528, PMID: 37760497 PMC10526929

[B23] Schubert J. P. Tay A. Lee K. H. C. Leong L. E. X. Rayner C. K. Warner M. S. . (2024). Genomic analysis of Helicobacter pylori in Australia: Antimicrobial resistance, phylogenetic patterns, and virulence factors. J. Gastroenterol. Hepatol. 39, 1869–1875. doi: 10.1111/jgh.16636, PMID: 38812101

[B24] Shang H. Wang T. S. Shen Z. Y. (2015). Quanguo Linchuang Jianyan Caozuo Guicheng (National Clinical Laboratory Operation Procedures). 4th edn (Beijing, China: People’s Medical Publishing House).

[B25] Tae Jun H. Nayoung K. Hong Bin K. Byoung Hwan L. Ryoung Hee N. Ji Hyun P. . (2010). Change in antibiotic resistance of *Helicobacter pylori* strains and the effect of A2143G point mutation of 23S rRNA on the eradication of *H. pylori* in a single center of Korea. J. Clin. Gastroenterol. 44, 536–543. doi: 10.1097/MCG.0b013e3181d04592, PMID: 20179610

[B26] Takeshi O. Hironori Y. Teruko N. In-Dal P. Myung-Woong C. Hideo Y. . (2002). A change in PBP1 is involved in amoxicillin resistance of clinical isolates of *Helicobacter pylori*. J. Antimicrob. Chemother. 50, 849–856. doi: 10.1093/jac/dkf140, PMID: 12461003

[B27] Tong Y. Dang R. Yin Y. Men C. Xi R. (2024). A whole genome sequencing-based assay to investigate antibiotic susceptibility and strain lineage of *Helicobacter pylori*. Microbial. pathogenesis. 197, 107069. doi: 10.1016/j.micpath.2024.107069, PMID: 39490594

[B28] Vincenzo D. F. Angelo Z. Enzo I. Dino V. (2009). Minimal inhibitory concentration (MIC) values and different point mutations in the 23S rRNA gene for clarithromycin resistance in *Helicobacter pylori*. Dig. Liver. Dis. 41, 610–6111. doi: 10.1016/j.dld.2009.01.001, PMID: 19200790

[B29] Wang X. Gong Y. He L. Zhao L. Wang Y. Zhang J. . (2023). Clinical relevance and distribution of *Helicobacter pylori* virulence factors in isolates from Chinese patients. Ann. Transl. Med. 11, 301–314. doi: 10.21037/atm-23-1404, PMID: 37181346 PMC10170277

[B30] Wang Y. Jiang T. Liu X. Sa R. Zhu X. Hu J. (2025a). Analysis of antibiotic susceptibility and genomic characteristics of *Helicobacter pylori* by whole-genome resequencing in Northern China. Braz. J. Microbiol. 56, 487–498. doi: 10.1007/s42770-024-01582-w, PMID: 39661273 PMC11885719

[B31] Wang Y. Zheng S. Guo R. Li Y. Yin H. Qiu X. . (2025b). Assessment for antibiotic resistance in *Helicobacter pylori*: A practical and interpretable machine learning model based on genome-wide genetic variation. Virulence 16, 2481503–2481516. doi: 10.1080/21505594.2025.2481503, PMID: 40119500 PMC11934168

[B32] Waskito L. A. Yamaoka Y. (2019). The story of *helicobacter pylori*: depicting human migrations from the phylogeography. Adv. Exp. Med. Biol. 1149, 1–16. doi: 10.1007/5584_2019_356, PMID: 31016625

[B33] Xue Z. Li W. Ding H. Pei F. Zhang J. Gong Y. . (2024). Virulence gene polymorphisms in Shandong *Helicobacter pylori* strains and their relevance to gastric cancer. PloS One 19, e0309844. doi: 10.1371/journal.pone.0309844, PMID: 39250512 PMC11383249

[B34] Zeng S. Kong Q. Wu X. Duan M. Nan X. Yang X. . (2024). Antibiotic resistance of *Helicobacter pylori* in Mainland China: A focus on geographic differences through systematic review and meta-analysis. Int. J. Antimicrob. Agents. 64, 107325–107329. doi: 10.1016/j.ijantimicag.2024.107325, PMID: 39245326

[B35] Zhang Y. Meng F. Jin J. Wang J. Gu B. Peng J. . (2021). Ninety-four thousand-case retrospective study on antibacterial drug resistance of *Helicobacter pylori*. World. J. Clin. Cases. 9, 10838–10849. doi: 10.12998/wjcc.v9.i35.10838, PMID: 35047595 PMC8678885

[B36] Zhong Z. S. Zhang Z. Y. Wang J. Hu Y. L. Mi Y. He B. S. . (2021). A retrospective study of the antibiotic-resistant phenotypes and genotypes of Helicobacter pylori strains in China. Am. J. Cancer. Res. 11, 5027–5037., PMID: 34765309 PMC8569369

[B37] Zhou W. Cheng H. Li M. Zhang R. Li Z. Sun G. . (2025). Effectiveness of susceptibility-guided therapy for *Helicobacter pylori* infection: A retrospective analysis by propensity score matching. Infect. Drug Resist. 18, 1149–1159. doi: 10.2147/idr.S498052, PMID: 40027915 PMC11871851

[B38] Zhou X. Lyu N. Zhu H. Cai Q. Kong X. Xie P. . (2023). Large-scale, national, family-based epidemiological study on infection in China: the time to change practice for related disease prevention. Gut 72, 855–869. doi: 10.1136/gutjnl-2022-328965, PMID: 36690433 PMC10086479

[B39] Zhou Y. Zhong Z. Hu S. Wang J. Deng Y. Li X. . (2022). A survey of antibiotic-resistant genotypes and strain lineages by whole-genome sequencing in China. Antimicrob. Agents Chemother. 66, e0218821. doi: 10.1128/aac.02188-21, PMID: 35652644 PMC9211431

[B40] Zhu M. Xu X. Cai P. Wang T. Zhu M. Yan C. . (2025). Global population structure, virulence factors and antibiotic resistance of *helicobacter pylori*: A pooled analysis of 4067 isolates from 76 countries. Helicobacter 30, e70025. doi: 10.1111/hel.70025, PMID: 40059062

